# Distinct short-range ovule signals attract or repel *Arabidopsis thaliana *pollen tubes *in vitro*

**DOI:** 10.1186/1471-2229-6-7

**Published:** 2006-04-05

**Authors:** Ravishankar Palanivelu, Daphne Preuss

**Affiliations:** 1 Department of Molecular Genetics and Cell Biology, The University of Chicago, Chicago, IL 60637, USA; 2 Howard Hughes Medical Institute, The University of Chicago, Chicago, IL 60637, USA

## Abstract

**Background:**

Pollen tubes deliver sperm after navigating through flower tissues in response to attractive and repulsive cues. Genetic analyses in maize and *Arabidopsis thaliana *and cell ablation studies in *Torenia fournieri *have shown that the female gametophyte (the 7-celled haploid embryo sac within an ovule) and surrounding diploid tissues are essential for guiding pollen tubes to ovules. The variety and inaccessibility of these cells and tissues has made it challenging to characterize the sources of guidance signals and the dynamic responses they elicit in the pollen tubes.

**Results:**

Here we developed an *in vitro *assay to study pollen tube guidance to excised *A. thaliana *ovules. Using this assay we discerned the temporal and spatial regulation and species-specificity of late stage guidance signals and characterized the dynamics of pollen tube responses. We established that unfertilized *A. thaliana *ovules emit diffusible, developmentally regulated, species-specific attractants, and demonstrated that ovules penetrated by pollen tubes rapidly release diffusible pollen tube repellents.

**Conclusion:**

These results demonstrate that *in vitro *pollen tube guidance to excised *A. thaliana *ovules efficiently recapitulates much of *in vivo *pollen tube behaviour during the final stages of pollen tube growth. This assay will aid in confirming the roles of candidate guidance molecules, exploring the phenotypes of *A. thaliana *pollen tube guidance mutants and characterizing interspecies pollination interactions.

## Background

**Figure 1:**
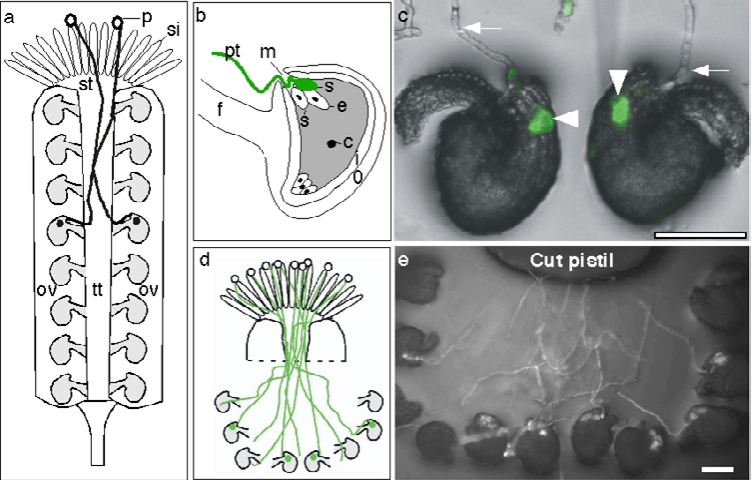
**Pollen tube targeting *in vitro***.(a) Diagram of a pollinated pistil within an *A. thaliana *flower. After reaching the stigma (si), pollen (p) extends a tube through the style (st) to reach the transmitting tract (tt) before entering one of the two ovary (ov) chambers to target an ovule. (b) Upon reaching the ovule, the pollen tube (pt, green) either grows up the funiculus (f) or makes a sharp turn towards the micropyle (m) and enters the ovule. Within the ovule, the pollen tube navigates towards the female gametophyte (gray) encased by outer (o) and inner (i) integuments, lyses within one of the two synergid (s) cells that flank an egg cell (e). Upon lysis, one sperm fertilizes the egg cell to form the zygote and the other fuses with the central cell (c) to form the endosperm. The number of pollen tubes drawn is for illustration purposes only and does not reflect the quantity typically observed in an assay. (c) Merged fluorescent and bright field images depicting the final stages of *in vitro *pollen tube growth. GFP-tagged pollen tubes make a committed turn (arrows) before entering a virgin ovule and lysing (arrow heads). (d) Diagram and (e) merged fluorescent and bright field image of *in vitro *pollen tube guidance assay. Pollen tubes emerge from the cut portion of the pistil, travel across the agarose medium before entering the excised ovules. Fluorescent green spot within ovules mark successful pollen tube targeting. Scale bars, 100 μm.

After a pollen grain lands on the surface of the pistil, it absorbs water from the stigma and forms a pollen tube – a long polar process that transports all of the cellular contents, including the sperm [1]. Pollen tubes invade the pistil and migrate past several different cell types, growing between the walls of the stigma cells, travelling through the extracellular matrix of the transmitting tissue, and finally arriving at the ovary, where they migrate up the funiculus (a stalk that supports the ovule), and enter the micropyle to deliver the two sperm cells-one fertilizes an egg and other the central cell (Fig. 1a, 1b) [2]. Typically, only one pollen tube enters the ovule through an opening called the micropyle, terminates its journey within a synergid cell, and bursts to release sperm cells-a process defined as pollen tube reception [3].

A combination of genetic and *in vitro *assays has defined signals that contribute to the early stages of pollen tube guidance. Chemocyanin, a small basic protein from lily stigmas, attracts lily pollen tubes *in vitro *[4], and in *A. thaliana*, wild type pollen guidance was abnormal when grown on stigmas over expressing the *A. thaliana *chemocyanin homolog [5]. Other signals are active in the nutrient-rich extracellular matrix secreted by the female transmitting tissue. A pectin that may promote guidance by mediating adhesion of pollen tubes to this matrix has been identified in lily [6]. Glycoproteins that likely contribute to guidance have also been described: in lily, a lipid transfer protein that contributes to adhesion [6], and in tobacco, two glycoproteins (TTS1 and TTS2) that provide nutritional and guidance cues are known [7,8]. Although potential homologs of these proteins exist in *A. thaliana*, their role in pollen tube growth is yet to be determined [6].

After emerging from the transmitting tract, pollen tubes approach the ovule micropyle with remarkable precision. Mutants defective in pollen tube guidance have demonstrated that this process is controlled by a series of molecular signals that involve pollen tubes, ovule tissues, and female gametophytes [1]. The *A. thaliana *mutants, *ino *[9] and *pop2 *[10] point to a role for diploid ovule tissue in pollen tube guidance; these have aberrant interactions between pollen tubes and diploid ovule cells, yet their female gametophytes appear normal, and in the case of *pop2*, can be fertilized with wild-type pollen [11]. Pollen tubes fail to either reach or enter the micropyle in *A. thaliana *mutants with nonviable or aberrant female gametophytes yet apparently normal diploid ovule tissue, providing strong support for the role of the haploid germ unit in promoting growth to the micropyle [12,13]. Based on these studies, it was proposed that final stages of pollen tube growth can be divided into two distinct phases: funicular guidance, in which pollen tubes adhere to and grow up the funiculus, and micropylar guidance, where pollen tubes enter the micropyle to deliver sperm to the female gametophyte [13]. Micropylar guidance signals originate at least in part from the two synergid cells contained within the female gametophyte; pollen tubes do not enter ovules in which synergid cells were either laser ablated [14] or defective due to a lesion in *A. thaliana MYB98 gene *[15]. The maize EA1 protein, which is exclusively expressed in the egg and synergids of unfertilized female gametophytes, may specify a role for these cells in regulating micropylar guidance. Plants expressing EA1 RNAi or antisense constructs produced significantly fewer seeds than wild type, and wild type pollen tubes failed to enter mutant ovules [16].

*In vitro *assays have been used to characterize intracellular cues such as a Ca^2+ ^gradient at the tip of pollen tubes that is critical for growth. Disrupting this gradient by iontophoretic microinjection or by incubation with Ca^2+ ^channel blockers can change the direction of tube growth [17]. The Ca^2+ ^gradient in pollen tubes is controlled by Rho GTPases; injection of antibodies against these proteins into pollen tubes, or expression of dominant-negative forms of RhoGTPase, causes the tip-focused Ca^2+ ^gradient to diffuse and eliminates tube growth [18], presumably by disrupting F-actin assembly [19]. These pollen tube growth defects can be partially alleviated by adding high concentrations of extracellular Ca^2+ ^[18].

*In vitro *grown pollen tubes also reorient their growth in response to certain extracellular cues; lily pollen tubes are attracted to chemocyanin [4] and repelled by a point source of nitric oxide [20]. In addition, *in vitro *grown pearl millet pollen tubes are attracted to ovary extracts [21]. For *T. fournieri *pollen tube guidance across a simple medium and into the ovule was achieved after pollen tubes were grown through a stigma and style [22]. In this species, the female gametophyte protrudes from the ovule, and pollen tubes enter the micropyle without interacting with funiculus [22]. Thus, the *T. fournieri in vitro *guidance system serves as a model for the micropylar, but not the funicular guidance phase of pollen tube growth to ovules [23]. Here, we describe an *A. thaliana in vitro *guidance assay that recapitulates both funicular and micropylar guidance, serving as a model for ovules with encased female gametophytes, an arrangement that is more common among flowering plants. With the sequenced *A. thaliana *genome, the large collection of mutants affecting reproductive functions, and comparative genomic resources, this assay will greatly facilitate identifying genes that mediate the final phases of pollen tube guidance.

## Results

### The *A. thaliana *stigma and style confer pollen tube targeting competence

Previous studies indicated that pollen tubes germinated in a simple growth medium cannot be guided to the micropyle [14,22]. Consistent with these observations, when such assays are performed for *A. thaliana*, few ovules are targeted (~3%, Table 1). Hence, we instead removed the upper portion of the pistil (the stigma and style [4,10,14,24]), deposited pollen on the stigma surface and showed that *A. thaliana *pollen tubes emerged from the style, travelled across an agarose medium to excised ovules and successfully entered the micropyle (Fig. 1a, 1b). To facilitate pollen tube observation, especially after they enter the micropyle and are obscured by the opaque ovule integument cells, we transformed plants with a GFP reporter under the control of the pollen-specific LAT52 promoter [25], and identified GFP-expressing lines with fully functional pollen tubes. Upon reaching the female gametophyte, these tubes burst and release a large spot of GFP (Fig. 1c, 1d, arrowheads and see Additional files 1 and 2), conveniently marking targeted ovules. Pollen tubes that grew within ~100 μm of an unfertilized ovule often made a sharp turn toward an ovule; of the tubes that grew within this range, ~50% successfully entered the micropyle (Table 1). This targeting efficiency is significantly higher than that of tubes germinated on agarose (Table 1). Thus, pollen tubes acquire the ability from pistil tissue to perceive ovule guidance signals, perhaps by absorbing essential nutrients or undergoing critical developmental transitions; a similar phenomenon was reported in *T. fournieri *[22]. In some cases, pollen grains that germinated on the stigma formed tubes that grew onto the medium, rather than penetrating the pistil and growing through the style. Nonetheless, these tubes successfully targeted excised ovules, suggesting that interaction with the stigma alone is sufficient to confer pollen tube guidance competence (Table 1); targeting efficiency was significantly reduced, however, suggesting that direct contact with female cells, rather than exposure to diffusible factors in the medium, is most important.

### Characterization of *A. thaliana *pollen tube-ovule interactions

**Figure 2:**
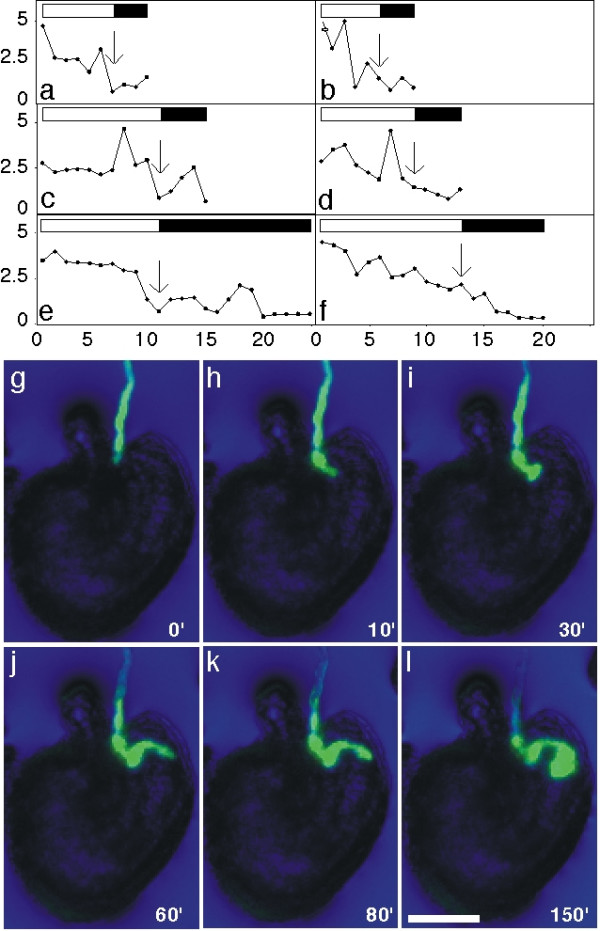
**Pollen tube navigation time course**. (a–f) Graphs of position (μm; X axis) versus growth rate (μm/min; Y axis) for six pollen tubes, from the moment they exit the style (white bar), enter the micropyle (arrow) and navigate within the ovule (black bar). (g–l) Merged fluorescent and bright field images of a GFP-tagged pollen tube entering a micropyle (g), navigating past diploid ovule cells (h–j), and pausing upon reaching a synergid (k), and completing lysis (l). Characterstics of pollen tube growth within the ovule shown in G-L were graphed and shown in (e). Time is min ('); scale bar, 50 μm.

As tubes left the style, they dispersed (Fig. 1e and see Additional File 1), growing at 2.5 ± 1.0 (s.d) μm/min (n = 20) and up to 3 mm before reaching an ovule (Fig. 2a–f). Near a virgin ovule, however, growth rates decreased (1.2 ± 0.59 (s.d) μm/min (n = 20;Fig. 2a–f) and the tubes often made sharp turns (Fig. 1d, arrows) within 33 ± 20 (s.d) mm of a micropyle (avg. 60 ± 38° (s.d); n = 60). Pollen tube guidance to ovules was abolished when fertilized or heat-treated ovules were used (Table 1), indicating they release a diffusible, heat-labile attractant prior to fertilization. Tube entry into the ovule appears to be not influenced by the number of tubes near a micropyle; targeting was achieved regardless of whether one or multiple tubes were in the vicinity of an ovule (see Additional files 1, 2, 3, 4). When approaching an ovule, the *in vitro *grown pollen tubes did not always migrate up a funiculus, Fig. 1c) before entering it. To directly test the role of this tissue, we removed funiculi from ovules, revealing a small but significant decrease in targeting efficiency (Table 1). These results indicate that an interaction between the pollen tube and funiculus is not essential, yet this interaction enhances successful entry into the ovule, perhaps by i) providing a physical support for pollen tubes to reach the micropyle, ii) aiding in the generation and maintenance of a signal gradient, or iii) enhancing the availability of ovule-derived guidance signals.

After responding to the attractant and entering an ovule, the growth rate of each pollen tube decreased to 0.98 ± 0.34 (s.d) μm/min (n = 19), reaching the female gamtophyte and lysing after a 73 ± 19 (s.d) min delay (n = 19; Fig. 2g–l and see Additional files 1 and 2). While previous work showed that pollen tube growth arrests only after reaching the female gametophyte [9,10], our observations point to additional signals that slow growth within the ovule prior to this arrest. This delay coincides with meandering pollen tube navigation past the integument and nucellus cells (Fig. 2g–k and see Additional files 1 and 2), potentially reflecting guidance by these cells.

### Short-range guidance signals from *A. thaliana *ovules are developmentally regulated

The data presented above indicate that contact with *A. thaliana *stigmas and styles enables pollen tubes to respond to diffusible ovule signals. To understand the nature and source of these signals, we examined their activity during ovule development. Previously it was shown that pollen tubes grow randomly or fail to elongate in immature *A. thaliana *pistils [26]. However, it was impossible to distinguish the contributions of distinct tissues in these experiments. Here, we exploited the modular nature of the *in vitro *system, varying the age of stigmas, styles, and ovules. While mature flower parts (stage 14 [27] were optimal, the stage of ovule development was critical, with guidance factors completely absent at ~32 hrs (stage 12a) and lower at ~16–24 hrs (stages 12b–c) before flowers mature (Table 2, upper panel). This pattern correlates with synergid development, the suggested source of pollen tube attractants; these cells form after stage 12a [27,28]. Even so, immature ovules promote better pollen tube guidance than heat-treated or fertilized ovules, suggesting that a basal signalling capability is established early and increases as the female gametophyte differentiates. In contrast, the developmental stage of the stigma and style did not significantly alter targeting (χ^2; ^P > 0.1; Table 2, bottom panel), indicating that the signals that confer targeting competence to pollen tubes do not vary over the course of pistil maturation (stages 12a–18) and that they emerge as early as stage 12a.

### Short-range guidance signals from ovules are highly species-specific

Like many traits that mediate reproduction [29,30], pollen tube guidance signals diverge rapidly – crosses between *A. thaliana *and its relatives show random or arrested pollen tube growth, even among species separated by <25 million years, MY [13,31]. Because these interspecies crosses utilized intact pistils, it has been impossible to discern the roles of individual tissues; moreover, early blocks in pollen tube migration have often made it difficult to assess interactions at downstream stages, including those near ovules. Here, we examined whether the stigma, style and ovule-derived signalling interactions are shared among *A. thaliana *relatives separated by ~5, 10, or 20 MY [31] (Table 3). The ability of the stigma and style to promote pollen tube competence was highly conserved (χ^2^; P > 0.05; Table 3, bottom panel), while the ovule-derived attractant diverged rapidly (χ^2^; P < 0.01; Table 3, upper panel). For example, *A. thaliana *pollen tubes inefficiently target ovules from *Arabidopsis arenosa *(separated by 5 MY from *A. thaliana*), rarely target *Olimarabidopsis pumila *ovules (10 MY), and fail to target *Capsella rubella *or *Sysimbrium irio *ovules (10 and 20 MY, respectively, Table 3, upper panel). Because *C. rubella *and *S. irio *are challenging to transform, it was not possible to test whether ovules from these two species are able to guide self-pollen expressing GFP under our assay conditions. Nonetheless, the ability of *A. thaliana *pollen to target ovules correlated with phylogenetic separation [30], suggesting that *A. thaliana *pollen tubes are sufficiently diverged that they fail to recognize attractants from *C. rubella *and *S. irio *ovules. Unlike the calcium signals that emanate from synergids [32,33], the proposed source of micropylar guidance signals, our results point to a diffusible, heat-labile ovule-derived signal that is sufficiently complex for rapid divergence – criteria that are most consistent with a protein-based signal. Pollen tubes perceive this signal at a distance of ~100μm from ovules after a 5 hour incubation in the assay. To estimate the molecular weight of this signal, we measured the diffusion rates of fluorescently-labelled dextran molecules under the same conditions in which the *in vitro *assay was performed, and calculated that the ovule-derived signal could measure up to approximately 85 kD (see methods).

### Targeted *A. thaliana *ovules repel supernumerary pollen tubes in vitro

**Figure 3:**
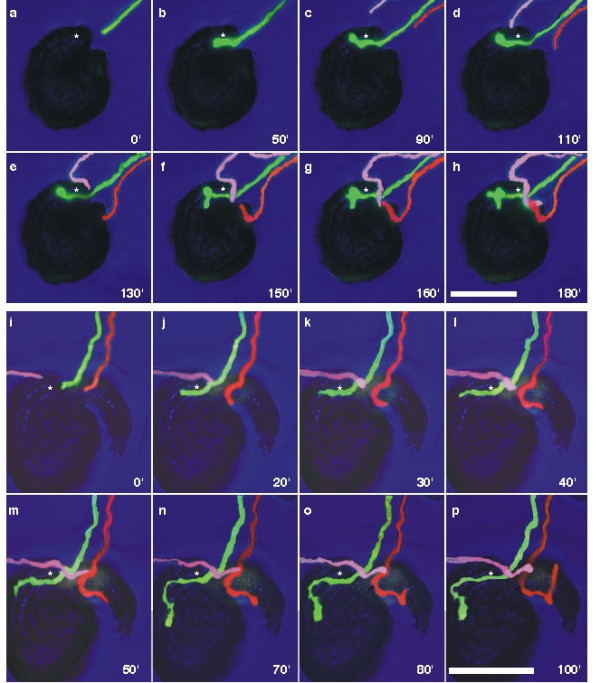
**Pollen tubes avoid targeted ovules**. Two examples (a–h and i–p) of pollen tube avoidance by ovules approached by three pollen tubes (false colour: green, the tube that enters the micropyle (*); pink and red, tubes that arrive later and do not enter; entire set of original images are presented in Additional Files 3 and 4). Elapsed time in minutes; Scale bars, 100 μm.

Interestingly, while the ovule-derived attractant in the *in vitro *assay acted to guide multiple pollen tubes toward ovules, only one pollen tube gained access to each micropyle. This is reminiscent of polyspermy blocks *in vivo*, where only one tube generally migrates up the funiculus and into the ovule [13]. While the mechanisms that prevent multiple tubes from even approaching an ovule are highly efficient, it is nonetheless possible for more than one pollen tube to enter a micropyle. In wild-type maize, heterofertilization results when the egg and central cell are fertilized by different pollen tubes at a frequency of ~1/50 [34] and in *A. thaliana*, ~1% (wild type) and ~10% (*feronia*) of ovules are penetrated by multiple pollen tubes [3]. When we performed the *in vitro *assay with fertilized ovules, many tubes grew within 100 μm, but none entered (Table 1), suggesting that the release of the ovule attractant terminates after fertilization, or alternatively, that a new signal repels additional pollen tubes. To distinguish between these possibilities, we used time-lapse imaging analysis (Fig. 3 and see Additional files 3 and 4). While 44% of targeted ovules (n = 143) were approached by additional pollen tubes, in every instance, these tubes did not enter the micropyle. Repelled tubes either stalled near the micropyle or turned sharply away from the targeted ovule (84 ± 42°; n = 61, Fig. 2e–h and see Additional files 3 and 4), a response that was observed as early as 10 min after a successful targeting event. This effect was fairly short-range; only tubes that approached within 27 ± 22 (s.d) μm were repelled. The diffusion rate of a series of dextran molecules through the medium used in this assay allowed us to estimate that a repellent measuring <10kD could diffuse ~27 μm in 10 minutes. Such abrupt turning behaviours were not observed when a single tube approached a virgin ovule. Instead, these tubes changed their growth direction by 60 ± 38° (s.d; n = 60), migrating toward, and not away, from the micropyle.

In the *A. thaliana *female sterile *feronia *and *sirene *mutants, wild type pollen tubes enter the mutant ovules but fail to cease growth or burst. In addition to this defect, multiple pollen tubes gain access to *feronia *and *sirene *mutant ovules [3,35]. Based on these results, it was suggested that repulsion of supernumerary tubes does not initiate until pollen tube reception occurs. Our observations with wild type pollen tubes in this *in vitro *assay indicate this is not the case – repulsion responses occurred well before tube growth terminated or tubes released their cytoplasm (n = 50, Fig. 3j, 3n and see Additional files 3 and 4). Moreover, while previous work suggested that female gametophyte cells release an inhibitory signal [3,35], our results show that repulsion initiated soon after the pollen tubes entered the micropyle and long before they reached the female gametophyte (Fig. 3 and see Additional files 3 and 4). Thus, this work points to a diffusible repulsive signal that is sufficient to override the ovule attractant. This signal may be derived directly from the diploid cells that surround the micropyle, from the female gametophyte, or from the successfully targeted, but unlysed, pollen tubes.

## Conclusion

Based on the results described here, we have defined three signaling events that regulate pollen tube guidance in *A. thaliana*: i) contact-mediated competence conferred by the stigma and style, ii) diffusible ovule-derived attractants and iii) repellents exuded from recently-targeted ovules. The species specificity and diffusion properties of the ovule attractant are consistent with a protein signal, while the abrupt transmission and response to the repellent suggests the activity of a small molecule, a peptide, or post-translational modifications to signals present before fertilization. This investigation also provides a platform to confirm the roles of candidate guidance molecules and to explore the phenotypes of *A. thaliana *mutants, including those that affect the development of diploid [9,10] and haploid [3,16,35] female tissues or pollen tubes [36]. The ability to characterize interspecies pollination interactions with this assay could lead to improvements in generating novel plant hybrids, a process that often requires *in vitro *manipulations [37].

## Methods

### Plant growth and material

Pollen was derived from LAT52:GFP transgenic lines (Columbia background). Stigmas, styles, and ovules were from the *A. thaliana *male sterile mutant, *ms1 *(CS75, Landsberg background) or from *A. arenosa *(CS3901), *O. pumila *(CS22562), *C. rubella *(CS22561) and *S. irio *(CS22653), deposited in the *A. thaliana *Biological Resources Center, Ohio State University. Female structures are unaffected by *ms1*, making it a convenient source of virgin pistils without the need for emasculation. No difference was detected between assays performed with materials derived from the Landsberg or Columbia ecotypes (not shown). Seeds were sown in soil and stratified at 4°C for 2 days, and plants were grown under fluorescent light (100 μE) for 16 or 24 hrs/day at 40% humidity. To consistently isolate pistils of varying developmental stages, we correlated the initial day of flowering of our plant population with previously defined floral development stages [26]. First, we confirmed that the youngest open flower is similar to stage 14 [26]. In *A. thaliana*, flowers continuously arise at the floral apex and are arranged in a spiral, with the younger buds on the inside. This predictable pattern allowed us to select stage 14 flowers as a starting point and identify older flowers (up to stage 18) and younger buds (up to 12a).

### *In vitro *pollen tube guidance assay

Growth medium for *in vitro *manipulations of pollen tubes [10] was determined to be optimal for also growing pollen tubes through a cut pistil. For the *in vitro *assays described here, pollen growth medium (3 ml) was poured into a 35 mm petri dish (Fisher Scientific, Hampton, USA). This volume of medium was ideal both for pollen tube growth and for microscopically viewing the interactions between pollen tubes and ovules. Excised pistils were pollinated under a dissection microscope (Zeiss Stemi 2000), cut with surgical scissors at the junction between the style and ovary (World Precision Instruments, Sarasota, USA), and placed horizontally on pollen growth medium. Pollen tubes emerged from the pistil ~3 hours after pollination and dispersed along the agarose surface for up to ~3 mm from the pistil.

Unlike previous reports [12,18], ovules were excised dry under a dissection microscope with a 27.5 gauge needle, from pistils that were held horizontally on double-sided tape (Scotch brand, 3M, St. Paul, USA). Excised ovules were immediately placed on the pollen growth medium, ~2 mm from the pistil, a distance that was typically accessible by the emerging pollen tubes. To maximize pollen tube-ovule interactions, 8–10 ovules were placed at the base of a pistil as shown in Fig. 1d. Because pollen tubes tend to disburse and grow randomly after leaving the style, not all ovules, particularly those placed near the cut pistil, are visited by a pollen tube (Fig. 1e).

For time-lapse imaging, ovules were placed with their micropylar end closest to the pistil excision site. Although not essential for targeting, ovules were oriented in this manner to reduce the time elapsed before targeting was achieved. *In vitro *assays were typically performed by completely coating stigmas of cut pistils with pollen (>100 grains per stigma); in contrast, for the repulsion assays only 20–30 pollen grains were deposited per stigma, making it possible to clearly observe individual tube behaviour. Based on experiments with limited amounts of pollen, we typically observed 50–80% of the pollen grains produced tubes that emerged from the style.

### LAT52:GFP transgenic plants

A HindIII fragment encoding GFP expressed from a post-meiotic, pollen-specific LAT52 promoter [25], was cloned into PBI121 (Clonetech, CA) and introduced into *A. thaliana *(Columbia) plants by *Agrobacterium*-mediated transformation. Kanamycin-resistant transgenic plants were selected, and a line containing a single transgene insertion, based on segregation of kanamycin resistance and GFP, was chosen for this study. This line had no detectable reproductive defects.

### Microscopy

Ovules targeted by pollen tubes in the *in vitro *assay were counted under a Zeiss fluorescent dissecting stereoscope. For calculating targeting efficiencies, we included only ovule micropyles within 100μm of a growing pollen tube; within this range pollen tubes exhibited responses that are typical for cells undergoing attraction: significant reorientation of growth towards the signal source, followed by a steady advance towards the target. For time-lapse fluorescent microscopy, GFP-labelled pollen tubes were observed using a Zeiss Axiovert 100 fitted with an automated shutter, motorized stage and CCD camera (CoolSNAP fxHQ, Roper Scientific, Inc Tucson, AZ). Images were captured at 10-minute intervals, converted to a TIFF format per the manufacturer's instructions using Slidebook (Intelligent Innovations Imaging, Santa Monica, CA). Pollen tube behaviours (growth rate, angle of turning, distance from micropyle) were measured and images were assembled into movies using ImageJ image analysis software (http://rsb.info.nih.gov/ij/download.html).

### Diffusion rates

To estimate the size of pollen tube signalling molecules, we performed time-lapse imaging of diffusion of a series of fluorescein-conjugated dextrans (Invitrogen, Carlsbad, CA) ranging in molecular weight from 3 to 70 kD on the pollen growth medium used for performing the *in vitro *pollen tube guidance assay. We dissolved each dextran compound in pollen growth medium, and spotted 2 ul each of 10 ng/ul and 100 ng/ul onto pollen tube guidance assay plates. Time-lapse imaging was performed as described for *in vitro *pollen tube behaviours except that images were captured once in 30 minutes. The rates of diffusion (3 kD = 5.5μm/min; 10 kD = 2.73 μm/min; 40 kD = 2.43 μm/min and 70 kD = 1.01 μm/min) were measured from these images using ImageJ software. Specifically, the fluorescent intensities along a line drawn from the centre of a dextran spot to the diffused periphery were calculated for an entire time-lapse series. The data was imported into Microsoft Excel and regression analyses were performed. Extrapolating from these values, molecular weights were estimated for attractants that would diffuse 33 μm and repellents that would diffuse 27 μm in 300 minutes (the time required for a typical pollen tube to reach an ovule in the *in vitro *assay).

### Statistical analysis

To measure the significance of the differences among observed ovule targeting efficiencies, we employed a χ^2 ^test for consistency in observed frequency distributions with a dichotomous classification and variable sample size [38].

## Authors' contributions

RP carried out experiments described in this study. RP and DP conceived, designed, coordinated this study and drafted the manuscript. Both authors read and approved the final manuscript.

## Supplementary Material

Additional File 1 ***In vitro *pollen tube guidance assay monitors ovule targeting by pollen tubes**. Time-lapse imaging of GFP-tagged pollen tubes emerging from the cut portion of the pistil, travelling across the agarose medium before approaching a subset of excised ovules. In this instance, pollen tubes grew within 100 μm only near two of the six ovules; of these two, only one was successfully targeted. Click here for file

Additional File 2 **Pollen tube migration within an ovule**. Time-lapse images showing a GFP-tagged tube entering and navigating within the ovule. The growth rate of the pollen tube decreases substantially before and soon after entry. Within the ovule, pollen tube ceases growth presumably in one of the synergids and tube discharge occurs, leaving a visible GFP spot. Click here for file

Additional File 3 **Pollen tube repulsion by a targeted ovule, example 1**. Three pollen tubes approach an ovule, however only one of them is ultimately successful. The unsuccessful tubes stall near the micropyle, despite coming very close to it. Click here for file

Additional File 4 **Pollen tube repulsion by a targeted ovule, example 2**. Three pollen tubes approach an ovule, however the pollen tube that arrived at the micropyle first was successful. The unsuccessful tubes approached the micropyle; however, one of them turned away from it, while the other one skips the micropyle and grows over the successful tube and stalls. The repulsion behaviors initiate well ahead of pollen tube discharge within ovule. Click here for file

## Figures and Tables

**Table 1:** *In vitro *ovule targeting efficiency

Female tissues				Fraction ovules targeted^a^	%	n^b^
			
Stigma	Style	Ovule	Funiculus			
–	–	+	+	4/162	3*	6
+	+	+	+	77/145	54	18
+	–	+	+	30/156	19*	25
+	+	+	–	61/175	35*	21
+	+	+ (fertilized)^c^	+	0/122	0*	20
+	+	+ (100°C)^d^	+	0/52	0*	8
+	+	+ (25°C)^d^	+	23/57	40	8

^a^Although the same number of ovules were placed in each assay, only those with a pollen tube tip ≤ 100 μm from the micropyle were considered potential targets; ^b^number of independent *in vitro *assays; ^c^expanded ovules, indicating embryo growth, were collected 1 day after pollination; ^d^ovules immersed in 100°C or 25°C water for 5 min; tissues present (+) or absent (–) after removal by microdissection (rows 1 and 4); assays in which pollen tubes germinated and grew only on the stigma (row 3); *, significantly different (χ^2^, *P *< 0.001) from assays performed with a stigma, style and intact funiculus (row 2).

**Table 2:** Developmental regulation of short-range guidance signals from ovules^a^

Developmental stage		Fraction ovules targeted^b^	%	n^c^
			
Stigma, style	Ovule			
14	12a	0/79	0**	13
"	12b	15/84	18**	7
"	12c	61/335	18**	31
"	13	51/159	32	18
"	14	195/398	49	41
"	15	50/120	42	18
"	16	48/119	40	15
"	17	47/104	45	15
"	18	40/103	39	13
12a	14	6/25	24	5
12b	"	14/43	33	8
12c	"	21/55	38	10
13	"	17/64	27	10
14	"	22/46	48	9
15	"	28/66	42	10
16	"	27/61	44	9
17	"	26/64	41	7
18	"	26/63	41	8

^a^Pollen grains were from *A. thaliana *stage 14 (*9*); ^b, c^as in ^a,b^Table 1; **, significantly different from assays with stigma, style and ovules from stage 14; (χ^2^) *P *< 0.001.

**Table 3:** Species specificity of short-range guidance signals from ovules^a^

Species		Fraction ovules targeted^b^	%	n^c^
			
Stigma, style	Ovule			
*A. thaliana*	*A. thaliana*	152/304	50	33
"	*A. arenosa*	50/161	31*	26
"	*O. pumila*	5/131	4**	21
"	*C. rubella*	0/87	0**	12
"	*S. irio*	0/107	0**	16
*A. arenosa*	*A. thaliana*	38/79	48	10
*O. pumila*	"	72/128	56	17
*C. rubella*	"	16/48	33	15
*S. irio*	"	17/36	47	12

^a^Pollen grains were from *A. thaliana *stage 14 (*9*); ^b,c^as in ^a,b^Table 1; *, **, significantly different from stage 14 *A. thaliana *assays in row 1; (χ^2^) *P *< 0.05, *P *< 0.001, respectively.

## References

[B1] Weterings K, Russell SD (2004). Experimental analysis of the fertilization process. Plant Cell.

[B2] Lord EM, Russell SD (2002). The mechanisms of pollination and fertilization in plants. Annu Rev Cell Dev Biol.

[B3] Huck N, Moore JM, Federer M, Grossniklaus U (2003). The Arabidopsis mutant feronia disrupts the female gametophytic control of pollen tube reception. Development.

[B4] Kim S, Mollet JC, Dong J, Zhang K, Park SY, Lord EM (2003). Chemocyanin, a small basic protein from the lily stigma, induces pollen tube chemotropism. Proc Natl Acad Sci U S A.

[B5] Dong J, Kim ST, Lord EM (2005). Plantacyanin plays a role in reproduction in Arabidopsis. Plant Physiol.

[B6] Mollet JC, Park SY, Nothnagel EA, Lord EM (2000). A lily stylar pectin is necessary for pollen tube adhesion to an in vitro stylar matrix. Plant Cell.

[B7] Cheung AY, Wang H, Wu HM (1995). A floral transmitting tissue-specific glycoprotein attracts pollen tubes and stimulates their growth. Cell.

[B8] Wu HM, Wong E, Ogdahl J, Cheung AY (2000). A pollen tube growth-promoting arabinogalactan protein from nicotiana alata is similar to the tobacco TTS protein. Plant J.

[B9] Baker SC, Robinson-Beers K, Villanueva JM, Gaiser JC, Gasser CS (1997). Interactions among genes regulating ovule development in Arabidopsis thaliana. Genetics.

[B10] Palanivelu R, Brass L, Edlund AF, Preuss D (2003). Pollen tube growth and guidance is regulated by POP2, an Arabidopsis gene that controls GABA levels. Cell.

[B11] Wilhelmi LK, Preuss D (1996). Self-sterility in Arabidopsis due to defective pollen tube guidance. Science.

[B12] Ray SM, Park SS, Ray A (1997). Pollen tube guidance by the female gametophyte. Development.

[B13] Shimizu KK, Okada K (2000). Attractive and repulsive interactions between female and male gametophytes in Arabidopsis pollen tube guidance. Development.

[B14] Higashiyama T, Yabe S, Sasaki N, Nishimura Y, Miyagishima S, Kuroiwa H, Kuroiwa T (2001). Pollen tube attraction by the synergid cell. Science.

[B15] Kasahara RD, Portereiko MF, Sandaklie-Nikolova L, Rabiger DS, Drews GN (2005). MYB98 is required for pollen tube guidance and synergid cell differentiation in Arabidopsis. Plant Cell.

[B16] Marton ML, Cordts S, Broadhvest J, Dresselhaus T (2005). Micropylar pollen tube guidance by egg apparatus 1 of maize. Science.

[B17] Gilroy S, Trewavas A (2001). Signal processing and transduction in plant cells: the end of the beginning?. Nat Rev Mol Cell Biol.

[B18] Zheng ZL, Yang Z (2000). The Rrop GTPase switch turns on polar growth in pollen. Trends Plant Sci.

[B19] Gu Y, Fu Y, Dowd P, Li S, Vernoud V, Gilroy S, Yang Z (2005). A Rho family GTPase controls actin dynamics and tip growth via two counteracting downstream pathways in pollen tubes. J Cell Biol.

[B20] Prado AM, Porterfield DM, Feijo JA (2004). Nitric oxide is involved in growth regulation and re-orientation of pollen tubes. Development.

[B21] Reger BJCRPR (1992). Chemotropic responses by pearl millet pollen tubes. Sexual Plant Reproduction.

[B22] Higashiyama T, Kuroiwa H, Kawano S, Kuroiwa T (1998). Guidance in vitro of the pollen tube to the naked embryo sac of torenia fournieri. Plant Cell.

[B23] Johnson MA, Lord E, Malho R (2006). Extracellular Guidance Cues and Intracellular Signaling Pathways that Guide Pollen Tube Growth. The Pollen Tube.

[B24] Cheung AY (1995). Pollen-pistil interactions in compatible pollination. Proc Natl Acad Sci U S A.

[B25] Twell D, Wing R, Yamaguchi J, McCormick S (1989). Isolation and expression of an anther-specific gene from tomato. Mol Gen Genet.

[B26] Kandasamy MK, Nasrallah JB, Nasrallah ME (1994). Pollen Pistil Interactions and Developmental Regulation of Pollen-Tube Growth in Arabidopsis. Development.

[B27] Christensen CA, King EJ, Jordan JR, Drews GN (1997). Megagametogenesis in Arabidopsis wild type and the Gf mutant. Sex Plant Reprod.

[B28] Christensen CA, Gorsich SW, Brown RH, Jones LG, Brown J, Shaw JM, Drews GN (2002). Mitochondrial GFA2 is required for synergid cell death in Arabidopsis. Plant Cell.

[B29] Swanson WJ, Vacquier VD (2002). The rapid evolution of reproductive proteins. Nat Rev Genet.

[B30] Ferris PJ, Pavlovic C, Fabry S, Goodenough UW (1997). Rapid evolution of sex-related genes in Chlamydomonas. Proc Natl Acad Sci U S A.

[B31] Hall AE, Fiebig A, Preuss D (2002). Beyond the Arabidopsis genome: opportunities for comparative genomics. Plant Physiol.

[B32] Mascarenhas JP, Machlis L (1962). Chemotropic Response of Antirrhinum majus Pollen to Calcium. NATURE.

[B33] Chaubal R, Reger BJ (1992). Calcium in the synergid cells and other regions of pearl-millet ovaries. Sexual Plant Reproduction.

[B34] Kato A (2001). Heterofertilization exhibited by trifluralin-induced bicellular pollen on diploid and tetraploid maize crosses. Genome.

[B35] Rotman N, Rozier F, Boavida L, Dumas C, Berger F, Faure JE (2003). Female control of male gamete delivery during fertilization in Arabidopsis thaliana. Curr Biol.

[B36] Johnson MA, von Besser K, Zhou Q, Smith E, Aux G, Patton D, Levin JZ, Preuss D (2004). Arabidopsis hapless mutations define essential gametophytic functions. Genetics.

[B37] Scholten S, Kranz E (2001). In vitro fertilization and expression of transgenes in gametes and zygotes.. Sexual Plant Reproduction.

[B38] Lister M (1993). 100 Statistical Tests.

